# Examination of scenarios introducing rubella vaccine in the Democratic Republic of the Congo

**DOI:** 10.1016/j.jvacx.2021.100127

**Published:** 2021-11-12

**Authors:** Alvan Cheng, Kurt Frey, Guillaume Ngoie Mwamba, Kevin A. McCarthy, Nicole A. Hoff, Anne W. Rimoin

**Affiliations:** a Department of Epidemiology, University of California, Los Angeles, CA, USA; b Institute for Disease Modeling, Bill & Melinda Gates Foundation, Seattle, WA, USA; c VillageReach, Ngaliema, Kinshasa, The Democratic Republic of the Congo

**Keywords:** Rubella, Congenital rubella syndrome, Vaccine introduction, Agent-based model

## Abstract

**Background:**

Rubella vaccine has yet to be introduced into the national immunization schedule of the Democratic Republic of the Congo (DRC); the current burden of congenital rubella syndrome (CRS) is unknown and likely to be high. An important consideration prior to introducing rubella containing vaccine (RCV) is the potential inverse relationship between RCV coverage and CRS incidence. Increasing RCV coverage will also increase in the average age of infection. Cumulative infections across all age groups will decrease, but the number of infections in age groups vulnerable to CRS may increase.

**Methods:**

Rubella transmission dynamics in the DRC were simulated using a stochastic agent-based model of transmission. Input parameter values for known properties, demographic variables, and interventions were fixed; infectivity was inferred from seropositivity profiles in survey data.

**Results:**

Our simulations of RCV introduction for the DRC demonstrate that an increase in CRS burden is unlikely. Continued endemic transmission is only plausible when routine immunization coverage is less than 40% and follow-up supplemental immunization activities have poor coverage for decades.

**Conclusion:**

Increased vaccination coverage tends to increase the annual variability of CRS burden. Simulations examining *low* vaccination coverage and high mean CRS burden are outbreak prone, with multiple years of reduced burden followed by acute outbreaks. These outcomes contrast simulations with *no* vaccination coverage and high mean CRS burden, which have more consistent burden from year to year.

## Highlights

• Serosurvey data suggest R_0_ values for rubella in the DRC on the range 3 to 8.
• Supplementary immunization activities provide multi-decade reduction in burden.
• Post-vaccine introduction, burden will likely be concentrated in outbreaks.

## Introduction

Global incidence of rubella has decreased significantly since the development of rubella-containing vaccines (RCV) in 1969 [1], [2]. Rubella is typically a mild disease in children but may be more severe in adults and can result in severe complications when pregnant women are infected. Infection during pregnancy may result in miscarriage, stillbirth, or a range of congenital abnormalities known as congenital rubella syndrome (CRS) [3], [4]. Transmission of rubella virus occurs through droplets from the respiratory tract, and infected individuals may be contagious from 7 days before to 7–12 days after rash onset [5]. Humans are the only known host for the virus, and there are no known animal reservoirs.

The most commonly used licensed rubella vaccines are based on the live, attenuated RA 27/3 strain and can be administered as either a monovalent formulation or as a combination with other vaccine viruses, such as measles, mumps, and varicella [1], [6]. A single dose of the rubella vaccine induces high seroconversion rates (≥95%) and provides long-term immunity, similar to that produced by natural infection [1], [7]. In most countries, RCV is administered along with measles vaccine and thus follows the two-dose schedule for measles: the first dose at 9 months or 12–15 months and the second dose at 15–18 months or 4–6 years [8], [9]. From 2000 to 2016, the number of countries administering RCV as part of their national immunization schedule increased by 54% (from 99 to 152 countries), and during this time period, the number of rubella cases reported to the World Health Organization (WHO) decreased by 97% from 670,894 cases in 102 countries to 22,361 cases in 165 countries [2]. The Democratic Republic of Congo (DRC) is a low-income country (LIC); rubella vaccine has yet to be introduced into the national immunization schedule of the DRC, and the burden of rubella and CRS is likely to be high. The country currently does not have a surveillance system for detecting rubella and CRS cases. Despite limitations in documentation, a recent study estimated that around 3000 infants are born with CRS annually in the DRC [10]. One-third of children 6- to 59-months and around 85% of women aged 15 – 46 years are seropositive for rubella antibodies [10], [11].

The primary goal of rubella immunization is to prevent congenital rubella and there are two general approaches of using RCVs: 1) focus solely on reducing CRS by immunizing only adolescent girls and women of childbearing age or 2) focus on interrupting transmission of rubella virus by immunizing all children through routine immunization (RI). Routine immunizations are vaccines recommended for all individuals based on their age and vaccine history. Research in the US, Israel, Japan, Iceland, and Norway has evaluated the economic impact of rubella-associated morbidities and the cost-effectiveness of rubella vaccination in these populations [12], [13], [14], [15], [16], [17]. It has been documented by Gudnadóttir and by Golden and colleagues that vaccination targeting specifically susceptible adolescent girls and women may be a more cost-effective strategy compared to vaccination of all children [14], [16]; however, with this approach, the epidemiology and circulation of rubella would likely remain unaffected as most infections occur before the age of immunization and elimination of CRS would likely not be achieved [1].

Another consideration prior to introducing RCV is the suggested inverse relationship between RCV coverage targeting all genders during childhood and CRS incidence. Previous mathematical models have suggested that this increase in CRS incidence may result from low coverage of RCV that increases the average age of infection [18], [19]. Many countries have introduced RCV, although recent evidence of this inverse behavior is limited. The WHO recommends that a country first achieve ≥ 80% coverage of the first dose of measles-containing vaccine (MCV1), through either RI or vaccination campaigns, before incorporating RCV into the national immunization schedule [20]. This caution has been amplified following instances where a reported rise in CRS cases have occurred following a period of low vaccination coverage, such as in Costa Rica and Greece [21], [22]. However, simulations of South Africa have suggested that in areas where the basic reproduction number (R_0_) for rubella is low, CRS incidence could be reduced even when vaccine coverage falls below the recommended level [23]. Based on these findings, incorporating rubella vaccination into the national immunization schedule of the DRC may provide an opportunity to reduce the burden of rubella and CRS; WHO and UNICEF estimates of national immunization coverage of MCV1 have persisted in the range from 60% to 70% over the past decade [24].

**Fig. 1:**
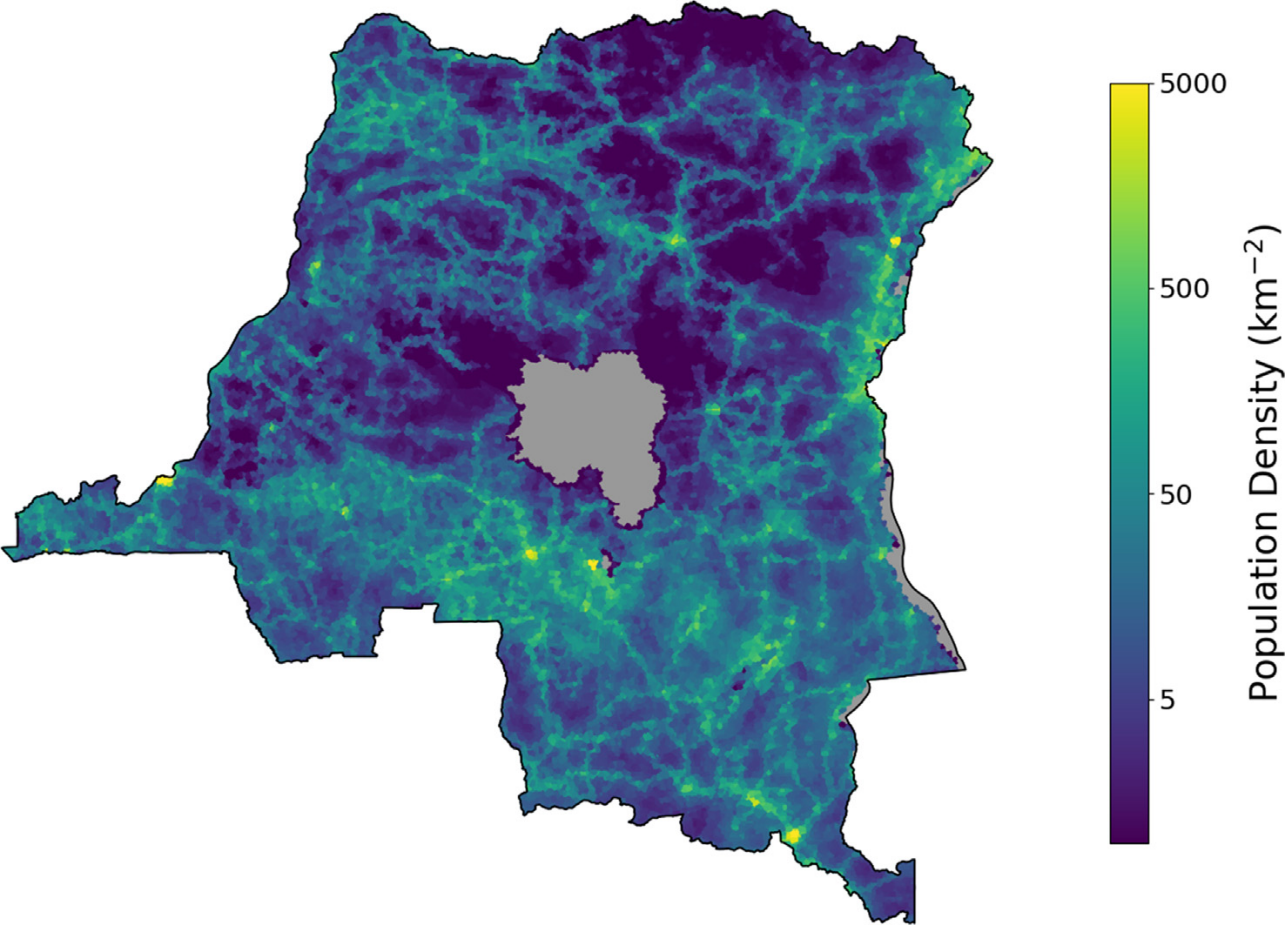
Visualization of estimated population density in the Democratic Republic of the Congo. Data from 2020 WorldPop estimates of UN adjusted, unconstrained population counts [45].

Despite the availability of an effective and affordable vaccine, rubella remains a leading cause of vaccine-preventable birth defects [2]. The Global Alliance for Vaccines Initiative (GAVI) has opened a funding window for rubella vaccination [25]. While the DRC does not currently provide RCV, it does offer the opportunity for two doses of measles vaccination through a combination of RI and supplementary immunization activities (SIAs). Taking advantage of these efforts to introduce RCV as a combined MR vaccine may provide an opportunity for progressing towards rubella and CRS elimination [26]. Health infrastructure in the DRC has struggled with limited roads and access to electricity and water; estimated population density is depicted in Fig. 1 and illustrates the challenging environment for nationwide coordinated vaccination. Maintaining and improving rates of routine immunization for measles has been difficult [27]. As rubella and measles immunization can be easily combined, there may be opportunity to leverage ongoing measles elimination activities to support rubella elimination.

We used an agent-based model to examine several different vaccination strategies for implementing rubella immunization to project outcomes for incorporating rubella vaccine into the national immunization schedule in the DRC. Mathematical models have been widely used in the management of infectious diseases and to assess the effectiveness of vaccination strategies [28], [29], [30].

## Methods

### Study population and design

Between November 2013 and February 2014, the second Demographics and Health Survey (DHS) was conducted in the DRC using a 2-staged stratified cluster design [31]. These nationally representative surveys collect data on maternal and child health, as well as basic demographic and health information [32], [33]. Information on sampling design and data collection procedures are described in more detail elsewhere [34]. Data were collected from a nationally representative sample of 18,171 households in which consenting women 15–49 years of age and men 15–59 years of age were eligible to participate in the survey. Data collected include, but are not limited to, demographics, health outcomes, and household composition.

In addition to survey data, dried blood spots (DBS) were also collected. Sample collection was primarily intended to inform HIV prevalence, but the Ministry of Health allowed for limited extension to measles, mumps, rubella, varicella, tetanus, malaria, and polio. Samples were collected in the field and stored with humidity indicator cards and desiccants to protect from molding and other events. Once the samples were received in Kinshasa, the lab team replaced the desiccant and humidity indicator cards and checked the quality of the samples. All were received in good condition. After the repackaging, samples were stored at −20 °C until testing. We stored the samples in high quality Bitran specimen bags separated by glassine paper. Testing of the samples started 15 months after collection and took 5 months to complete. The humidity indicator cards were monitored during the sample testing to ensure that samples were kept in good condition. All samples were kept at −20 °C during laboratory analysis and only removed when tested. Survey data from paper questionnaires were converted to an electronic format using the Census and Survey Processing System (US Census Bureau, ICF Macro, Rockville, MD) and were double entered and checked for inconsistencies by comparing the two datasets. Ethical approval was obtained at UCLA Fielding School of Public Health, the Kinshasa School of Public Health, and the Centers for Disease Control and Prevention.

### Laboratory analyses

DBS samples were collected using a modified extraction protocol and analyzed at the UCLA-DRC laboratory located at the National Laboratory for Biomedical Research in Kinshasa, DRC [35]. Laboratory testing was completed using the Dynex multiplex Measles, Mumps, Rubella, Varicella, and Tetanus (MMRVT) immunoassay platform [36]. Quarter inch DBS samples were eluted out in phosphate buffered saline containing 0.05% Tween-20 and 5% dried milk, equating to a 1:143-fold dilution assuming 7 μl of serum per DBS sample. Polystyrene beads coated separately with antigen to measles, mumps, rubella, varicella-zoster virus, and tetanus were immobilized within 54-well assay strips with 10 beads per well and processed using the Dynex system for IgG antibody detection. Based on epidemiologic studies, the positive/negative cutoff for rubella IgG antibody detection used in our analyses was set at 10 IU/mL [37], [38].

### Epidemiological model

Rubella transmission dynamics in the DRC were simulated using the Generic branch of EMOD, a stochastic agent-based model of transmission [39]. Input parameter values for known properties (e.g., infectious period), demographic variables (e.g., birth rate), and interventions (e.g., routine immunization) were fixed; latent quantities (e.g., R_0_) were inferred based on the seropositivity profiles from the survey data. A constant low-level of importation ensured that re-introduction was possible in the event of local elimination. See Additional File 1 for details on model implementation.

### R_0_ estimation

Infectivity, the measure of transmission potential of an infectious agent, can be characterized by a basic reproductive number (R_0_), which describes the mean number of secondary infections that a single primary infection would generate in a totally susceptible population [28]. This property is a consequence of both the biology of the pathogen and the social network structure of the at-risk population. Accurate estimation of R_0_ is important because it also describes the immunity level necessary to control the spread of infection with that population [40].

Simulations of each province generated age-stratified seronegativity profiles of the population over a range of R_0_ values from 1.5 to 15.0. These profiles were compared to the observed profiles collected during the DHS sampling period, which informed posterior distributions of infectivity used for CRS burden projections. See Additional File 2 for details on the estimation of R_0_.

### Burden forecasts

Age-structured infection time-series for the DRC provinces were simulated for a three-decade period, intended to correspond to the years 2021 to 2050. Outcomes for each province were independent. The value of R_0_ in each province for each of these simulations was drawn randomly from the posterior distributions previously described. These time-series of infections were converted into occurrences of CRS using fixed probabilities for each age group; results are presented as rates based on simulated population or simulated births where noted. See Additional File 2 for details on the calculation of CRS burden.

## Results

### R_0_ estimation

**Fig. 2:**
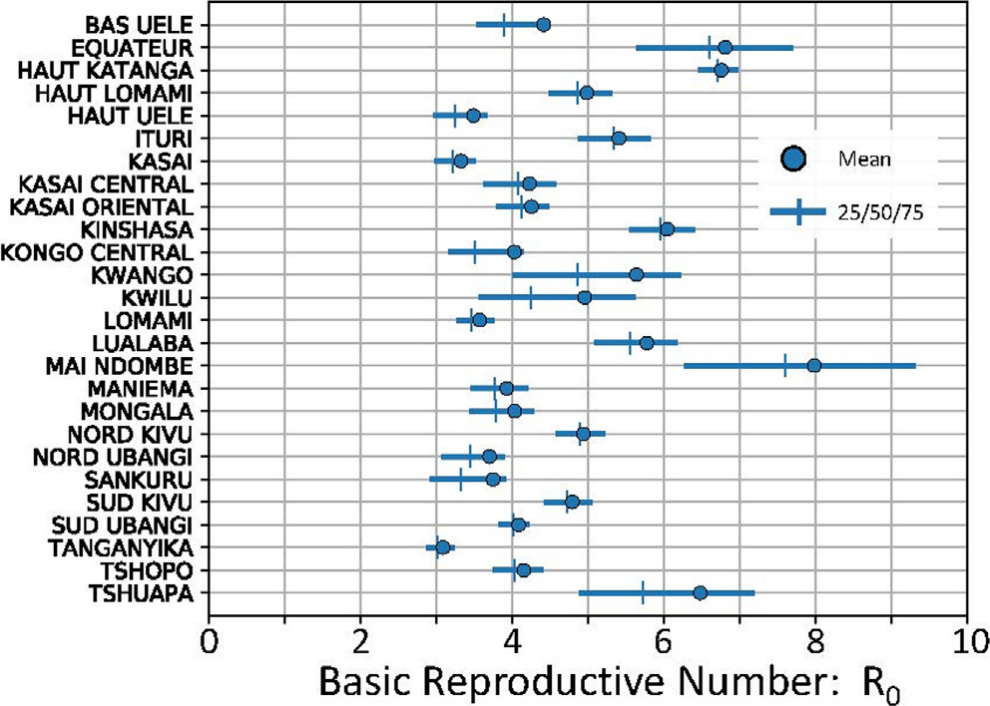
Summary statistics for posterior distributions from simulation outcomes estimating rubella infectivity in the provinces of the DRC. Mean values are depicted as circles; quartile values are depicted as solid lines with vertical mark at the median.

Values for the estimated R_0_ varied by province; mean estimates in each province were on the range from 3 to 8. Summary statistics for the outcome distributions are depicted in Fig. 2. These posterior distributions were used to inform transmission dynamics for the burden estimates. Distributions tended to be positively skewed, reflecting that history of infection was present in all communities and values of R_0_ less than unity were not possible under the model structure used.

Most posterior distributions were unimodal, although the distribution for the province of Mai Ndombe was bimodal. The two peaks correspond to two transmission regimes within the model: endemic and outbreak driven. Endemic transmission tends to sustain non-zero, although not necessarily constant, levels of prevalence throughout a simulation; outbreak driven transmission involves variable length periods of zero prevalence, with importations that may lead to epidemic behavior. Serosurvey data are not necessarily able to independently distinguish between these two regimes. A single seronegativity profile can correspond equally well to an endemic, highly infectious pathogen, or proximity to an outbreak of a pathogen with low to moderate infectivity [41]. Although Mai Nodmbe was the only province to have a clearly bimodal infectivity posterior, other provinces with diffuse outcomes (e.g., Equateur, Tshuapa) also exhibit this ambiguity.

### Baseline CRS estimation

Estimated burden in each province over the period 2021 to 2050 was independent of year when scaled by total population. The demographics model used in the simulations assumed a constant population growth rate (province dependent; between 2.4% and 4.0% as detailed in Additional File 1) and no changes to the shape of the population pyramid; with no other time varying quantities (i.e., burden forecasts in this base model did not include vaccination), this invariance with respect to time represents correct implementation of the model structure.

**Fig. 3:**
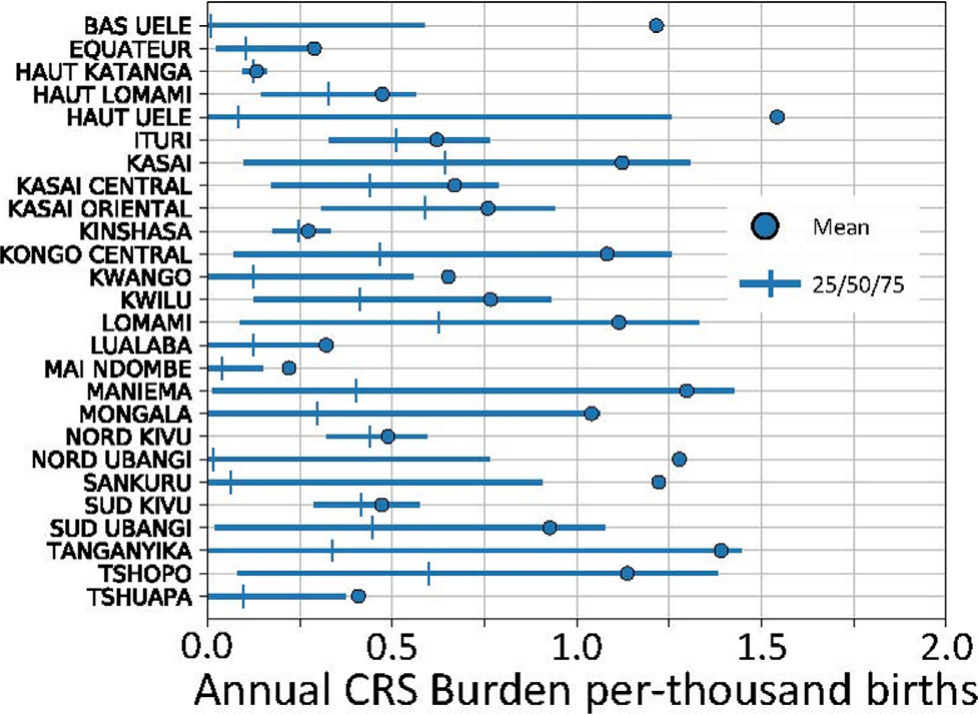
Summary statistics for posterior distributions from simulations estimating burden of congenital rubella syndrome in the provinces of the DRC. Mean values are depicted as circles; quartile values are depicted as solid lines with vertical mark at the median.

Values for the estimated annual burden of CRS per-thousand-births varied by province; mean estimates in each province were on the range from 0.1 to 1.6. Given a crude birth rate of around 40-per-1000, and a total population of around 90 M, an annual rate of 1.0 per-thousand-births corresponds to about 3600 cases of CRS annually, which is similar to other estimates of pre-vaccine disease burden [42]. Summary statistics for the outcome distributions are depicted in Fig. 3.

Distributions tended to be very positively skewed, with median burden of less than 0.1 per-thousand-births in several provinces. The skewness is a consequence of the degree that outbreak dynamics tend to drive total burden. More populous provinces (e.g., Kinshasa, Nord Kivu, Haut Katanga) did not have notable skew in annual outcomes and were associated with mostly endemic transmission. Endemic transmission was also associated with lower total mean CRS burden. This lower burden was due to a younger average age-at-infection, suggesting higher levels of naturally acquired immunity prior to childbearing age.

### Scenario CRS estimation

Introduction of RCV was simulated as occurring via a catch-up SIA targeting individuals with age 9 months to 15 years, followed by inclusion in the routine immunization schedule as part of single dose measles regimen (a transition of M to MR without adjustment of timing or coverage), and the use of MR vaccine in all follow-up SIAs (targeting ages 9 months to 5 years). No increase in RI coverage was assumed in any scenario. Several levels were examined for the effective coverage of the catch-up SIA campaign, along with coverages and frequency of follow-up SIA campaigns. Here, effective coverage is defined as the fraction of the target population receiving immunity from the campaign.

**Fig. 4:**
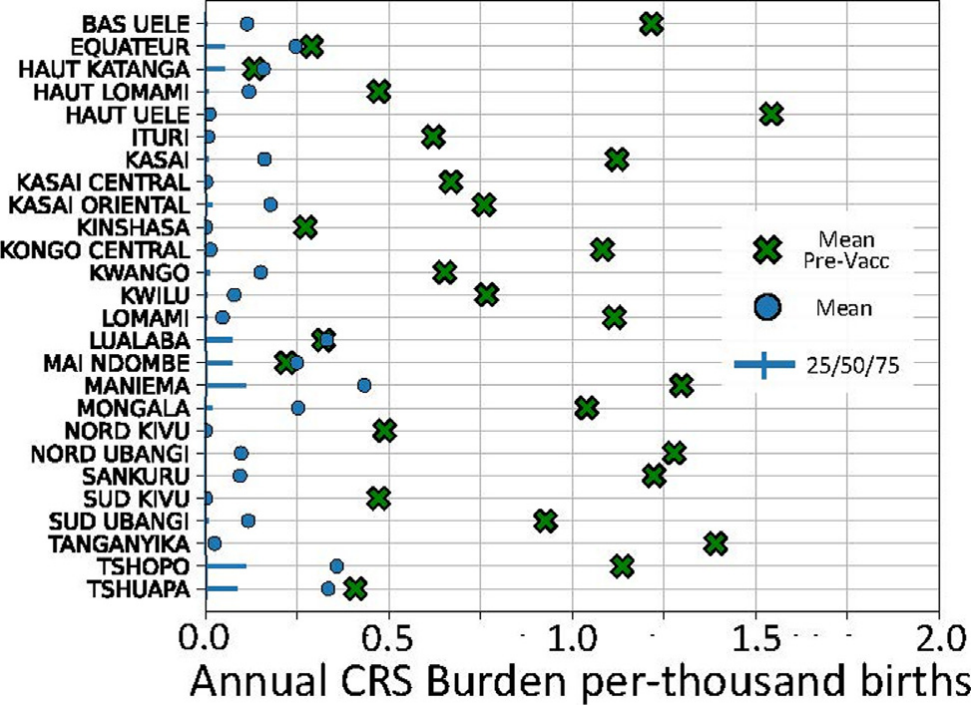
Summary statistics for posterior distribution from simulations estimating burden of congenital rubella syndrome in the provinces of the DRC following introduction of RCV in RI, a catch-up SIA, and subsequent follow-up SIAs every four years. Coverage of all SIAs was implemented at 50%. Mean values are depicted as circles; quartile values are depicted as solid lines with vertical mark at the median. Mean values from Fig. 3 (equivalent simulations without vaccination) are included as green crosses for reference. (For interpretation of the references to colour in this figure legend, the reader is referred to the web version of this article.)

Fig. 4 depicts the expected CRS burdens in the most pessimistic scenario, which used 50% coverage for all SIAs and one-per-four-years frequency of follow-up SIAs. This scenario would not be expected to provide adequate control of measles transmission and was chosen as a plausible lower bound for rubella vaccine usage post introduction. In this scenario, as in all scenarios examined, the median annual burden decreased to near zero. However, this scenario was noteworthy because the mean annual burden did not decrease for all provinces; in Equateur, Haut Katanga, Lualaba, Mai Ndombe, and Tshuapa the mean burden remained roughly the same but was differently distributed. For these provinces in this scenario, incidence was concentrated in infrequent but large epidemics occurring after reintroduction following local elimination.

### Hypothetical CRS estimation

An artificial context was created using mean provincial birth and growth rates; these rates are detailed in Additional File 1. Infectivity in this context was sampled from a posterior distribution with mean R_0_ of 5. Simulations using this context introduced RCV though the routine immunization schedule only and did not implement a catch-up SIA or use RCV as part of follow-up SIAs. All levels of routine immunization coverage were examined; these levels were static and did not change over time post vaccine introduction. These simulations depict the potential of inverse CRS response at vaccination levels lower than around 65%, but also emphasize the time-variation of burden in response to vaccine introduction.

**Fig. 5:**
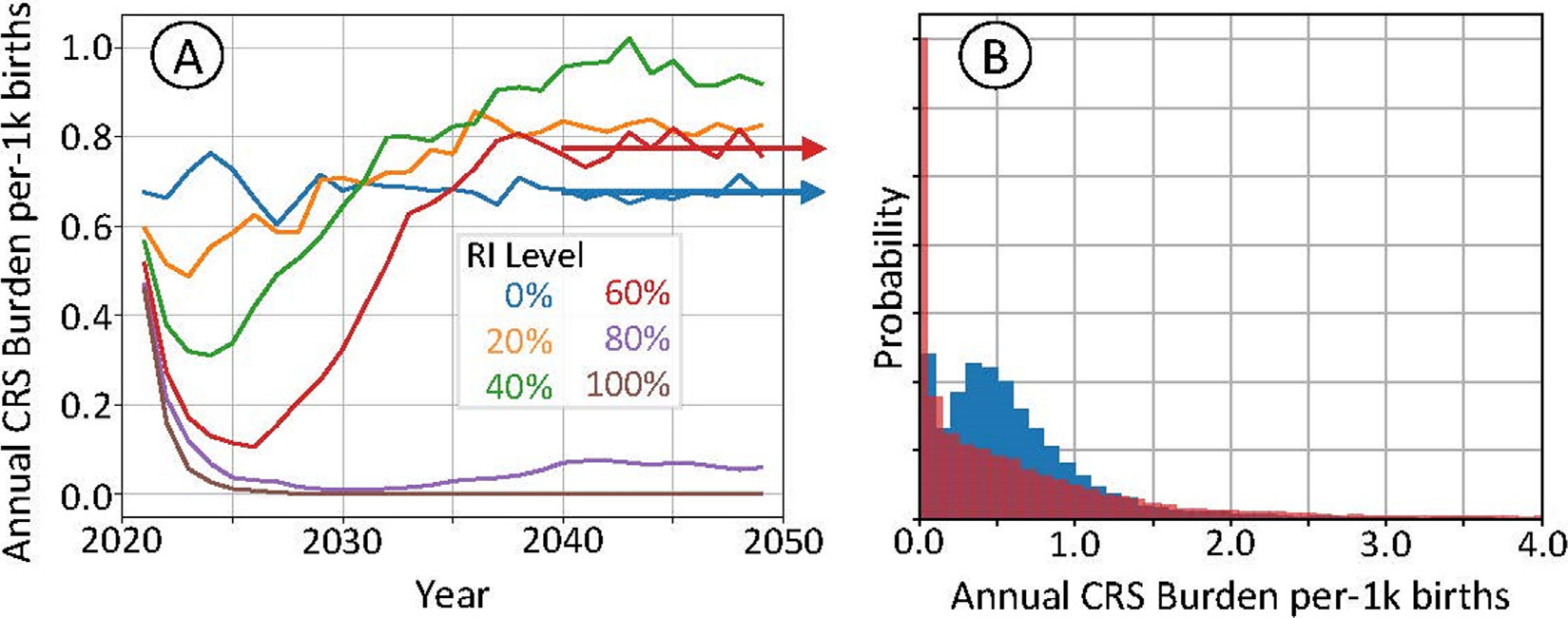
(A): Mean annual CRS burden per thousand births as a function of time following RCV introduction through routine immunization only with no SIAs; outcomes for a hypothetical context using an R_0_ of around 5. (B): Histograms of the annual CRS burden per thousand births in this context for the period 2040 to 2050; distributions for simulations at the 0% (blue) and 60% (red) levels of RI. (For interpretation of the references to colour in this figure legend, the reader is referred to the web version of this article.)

Fig. 5a depicts the change in CRS rates over the 30-year simulation window for the full range of routine immunization coverages; the 0% coverage level provides a reference trajectory. Coverages are taken as effective coverages: the fraction of the target population receiving immunity through vaccination. All trajectories with vaccine demonstrate an initial decrease in CRS burden post vaccine introduction, although equilibrium rates do not fall below the no-vaccine scenario until routine immunization coverage is above about 65%. Fig. 5b depicts histograms of simulated CRS burden over the period 2040 to 2050 for the 0% and 60% coverage levels; this period was selected so the distribution would be near equilibrium. Although both scenarios have similar levels of CRS burden, simulations with vaccine have created greater variance in outcomes. Annual CRS burden for the 60% coverage level is likely to be either large or near-zero, with near-mean outcomes occurring infrequently.

Additional simulations using this artificial context in conjunction with SIAs are presented in Additional File 4.

## Discussion

Estimates of rubella infectivity from serosurvey data tended to vary on the range from 3 to 8 and fell into two distinct regimes: endemic or epidemic. Serosurvey profiles assuming endemic, or roughly equilibrium, rubella transmission correlated with greater infectivity. These levels were not plausible for several provinces where endemic rubella implied R_0_ > 12, suggesting that the sampling frame of the serosurvey occurred within a few years of a significant rubella outbreak.

Simulations that do not incorporate SIAs can result in increased CRS burden following the introduction of RCV, although this increase in burden is a long-term phenomenon and the short-term impact of RCV introduction is a decrease in burden for a period of several years. Each province was approximated as well mixed and independent, which was appropriate given the serosurvey data available. See Additional File 3 for details on sub-provincial heterogeneity. Although the data do not demonstrate R_0_ sub-provincial heterogeneity, this result is likely a consequence of the sparsity of the serosurvey results. Actual within province heterogeneity is very high with many communities only accessible via river, and remote communities would be expected to correspond to lower values of R_0_.

The addition of SIAs qualitatively changes expectations for CRS burden. No increase in burden was estimated for any introduction scenario; all scenarios resulted in a decrease in median annual CRS. In the most conservative scenario examined, an SIA every fourth year that achieves around 50% coverage, simulations did not result in an increase in burden. By targeting the 9-month to 5-year old demographic every fourth year, this scenario increased overall effective vaccine coverage by around 50%. This increase in coverage was sufficient to prevent an increase in burden, although the mean CRS burden for several provinces did remain roughly the same. Provinces where burden was not reduced were characterize by having high estimates of rubella infectivity (R_0_ > 6). Equilibrium CRS rates for the artificial context with no SIAs fall below the no-vaccine scenario when routine immunization coverage increases above 65%. This threshold will vary based on infectivity, birth rate, and other factors; it is not intended to represent a fixed threshold for rubella vaccine usage. Internal and international migration were also not addressed by these simulations. Susceptible people displaced internally to the DRC or across one of its borders could increase the likelihood of an outbreak during the intra-SIA period. Reliance on SIAs to compensate for gaps in routine coverage is not a viable long-term solution and is particularly vulnerable to disruptions.

Simulations of increased vaccination coverage also resulted in greater variance in CRS burden, consistent with phenomena described in the canonical path to elimination for measles [43]. The implication of this behavior is that at elevated levels of RCV coverage the burden tends to be concentrated in infrequent epidemic occurrences rather than persistently elevated levels of incidence. In these situations, preemptively identifying and addressing gaps in immunity prior to epidemic occurrence would prevent much of the burden. Outbreak control is also very important programmatically to ensure confidence in immunization and health policy [44]. Increased epidemic behavior may potentially erode trust in vaccination services even when absolute total burden is decreased. Strengthening rubella surveillance enables improved outbreak control; it complements strengthening measles surveillance and is central to improved public health services.

## Declaration of Competing Interest

The authors declare that they have no known competing financial interests or personal relationships that could have appeared to influence the work reported in this paper.
