# Corrigendum to “Examination of scenarios introducing rubella vaccine in the Democratic Republic of the Congo” [Vaccine: X 9 (2021) 100127]

**DOI:** 10.1016/j.jvacx.2022.100215

**Published:** 2022-09-16

**Authors:** Alvan Cheng, Kurt Frey, Guillaume Ngoie Mwamba, Kevin A. McCarthy, Nicole A. Hoff, Anne W. Rimoin

**Affiliations:** a Department of Epidemiology, University of California, Los Angeles, CA, USA; b Institute for Disease Modeling, Bill & Melinda Gates Foundation, Seattle, WA, USA; c VillageReach, Ngaliema, Kinshasa, The Democratic Republic of the Congo

The authors regret age-specific fertility rates were incorrectly applied when determining burden of congenital rubella syndrome (CRS). Annual fertility rates, averaged over 3 years, were used as rate-*per*-3-years. Estimated CRS burden in all scenarios should be 3 times greater than indicated.

Corrected scales for the x-axis in Figs. 3 and 4 should be 0–6 (instead of 0–2 as depicted). Corrected scale for the y-axis in Fig. 5a should be 0–3 (instead of 0–1 as depicted), and the corrected scale for the x-axis in Fig. 5b should be 0–12 (instead of 0–4 as depicted).

**Fig. 3:**



**Fig. 4:**



**Fig. 5:**



The authors would like to apologise for any inconvenience caused.

