# Supplementary material for: Examination of scenarios introducing rubella vaccine in the Democratic Republic of the Congo
Source: Vaccine X. 2021 Nov 12;9:100127. doi: 10.1016/j.jvacx.2021.100127 (PMC8608602; doi:10.1016/j.jvacx.2021.100127)
Supplement: Supplementary data 1 [file mmc1.pdf]

Examination of scenarios introducing rubella vaccine in the Democratic Republic of the Congo

Additional File 1 - EMOD Software Details

Kurt Frey

The Institute for Disease Modeling (IDM) is committed to improving and saving lives in developing countries through the use of quantitative analysis. Its primary software, Epidemiological Modeling (EMOD), is a stochastic agent-based model of disease transmission. The Generic branch of this software is not specific to any disease, and was used to represent rubella by selecting rubella-appropriate parameter values.

Overview

A single EMOD simulation follows a collection of agents through an arbitrary number of discrete time steps. Simulations in this study used a constant length time step constructed to represent one day. Years were approximated as 365 time steps; leap days were neglected. Simulations used for estimating the base reproductive number (R_0) had a duration of 51 years, which was taken to represent the period from 1970 to 2020; simulations used for estimating the burden of congenital rubella syndrome (CRS) had a duration of 81 years, which was taken to represent the period from 1970 to 2050.

Simulation results for the period 1970 to 2010 were not intended to be an accurate historical reproduction. Those time steps were used to provide an equilibration period so that the period from 2011 onward would not be biased by the choice of initial conditions.

Each agent was assigned to one of 26 nodes, representing the current provinces of the Democratic Republic of the Congo (DRC). The total population for each province in 2019 was calculated using health zone populations from the DRC health zone shapefile on the Humanitarian Data Excahnge (HDX), hosted by the United Nations Office for the Coordination of Humanitarian Affairs [3] (UN-OCHA). Population growth rates within the simulation were maintained at a constant value; these values are included in table 1 along with total populations and province names. Growth rate calculations are described as part of the Demographics section.

An overall flow diagram representing the different health conditions and transitions present for each individual in the disease model is presented in Figure 1. Note that EMOD is agent-based; health conditions are descriptive and do not represent homogeneous compartments. Descriptions of the Immunity and Disease processes are described in their respective sections.

Table 1: Nodes, populations, and growth rates within the EMOD simulations.

Country	Province	Est. 2019 Population	Annual Growth (%)
DRC	Bas Uele	1177029	2.746
	Equateur	2247840	3.278
	Haut Katanga	5440816	3.644
	Haut Lomami	3632535	3.644
	Haut Uele	1701503	2.746
	Ituri	5226511	2.746
	Kasai	4099745	3.979
	Kasai Central	4369364	3.979
	Kasai Oriental	4882512	3.396
	Kinshasa	8647308	2.475
	Kongo Central	3635569	3.084
	Kwango	2294566	3.126
	Kwilu	4592343	3.126
	Lomami	3600553	3.396
	Lualaba	2090404	3.644
	Mai Ndombe	1762664	3.126
	Maniema	2391851	2.707
	Mongala	2314755	3.278
	Nord Kivu	8783179	3.073
	Nord Ubangi	1452136	3.278
	Sankuru	1846756	3.396
	Sud Kivu	6445549	3.529
	Sud Ubangi	2636370	3.278
	Tanganyika	2815857	3.644
	Tshopo	2965751	2.746
	Tshuapa	1945663	3.278

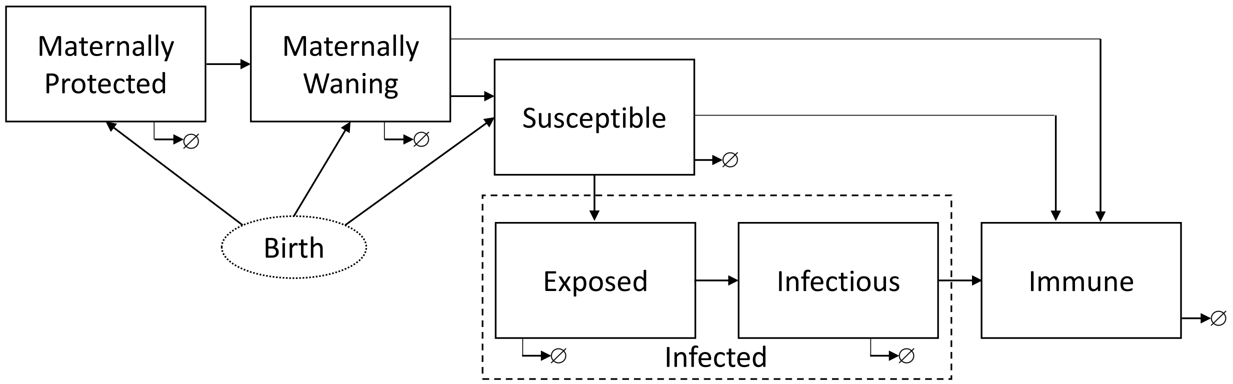

Figure 1: Flow diagram representing the health conditions and transitions present in the disease model. Agents are born as either maternally protected, maternally waning, or susceptible. Mortality is possible for all conditions and unrelated to disease status. Note that there is no transition from maternally protected or exposed directly to immune. Vaccination that occurs while an agent is maternally protected, or in an infected condition has no effect and is assumed to not provide immunity.

Demographics

Birth (addition of new agents), aging (incrementing age of agent), and mortality (removing agents) occurred for each time step in the simulation. Birth rates and mortality probabilities are listed in Table 2.

Birth

The birth process added new agents (age = 0 days) to each node in proportion to the current number of agents present in that node at the start of the time step. Agents were added subsequent to the aging process, so new agents did not age until the following time step. The per-day birth rate for a node was used as a rate in a Poisson distribution, and the number of new agents added to the simulation during that time step was randomly drawn from that distribution. Values in Table 2 were estimated based on the 2013 DRC Demographics and Health Survey (DHS) [1].

Mortality

Agents were removed from the simulation at the end of each time step with an age dependent probability given in Table 2. Probabilities for the age ranges 0 - 29 days, 30 - 359 days, and 360 - 1829 days were calculated using data from the 2013 DRC DHS [1] and correspond to neonatal mortality, post-neonatal mortality, and child mortality. Probabilities applied to agents with ages $\geq$ 1830 days were adjusted to provide a national population pyramid in line with DHS estimated pyramid for 2013.

This mortality process was the only method for agents to be removed from the simulation. Although EMOD supports disease-specific agent mortality, that process was not included for this study. These demographic processes were intended to create a representative distribution of ages so that the reported age-at-infection could be used to inform disease transmission dynamics. Probabilities in Table 2 are interpretable as all-cause mortality, but were not used when calculating disease-associated burden.

Population

Given the time invariant birth and mortality values in Tables 2, the age distribution of agents (i.e., population pyramid) was asymptotically stable. Initial agent ages for each simulation were drawn from an equilibrium distribution of ages. Although actual birth and mortality rates are time varying quantities, this initialization procedure guaranteed that the population pyramid matched reported values for the year 2013 and included accurate mortality rates for children $\leq$ 5 years old. It was assumed that this profile changed slowly enough to provide a satisfactory approximation for the period 2000 to 2020.

The total number of agents grew at a rate with constant mean value (i.e., constant population growth rate), which was a consequence of time invariant birth and morality rates. Initial numbers of agents for each state were selected so that the expected population in 2019 was equal to the values given in Table 1.

No permanent migration was included in these simulations. The trend toward urbanization would contribute toward a likely underestimate of the future populations of urban provinces such as Kinshasa. The assumption of a constant growth rate was one of the greatest source of uncertainty in this model.

Table 2: Input parameters for birth and mortality processes. The birth rate is given in this table per-thousand, per-year; mortality values are the age-varying daily probability of an agent being removed.

Province	Birth Rate	Mortality				
		Day 0 - 29	Day 30 - 359	Day 360 - 1829	Day 1830 - 34679	Day 34680 -
Bas Uele	39.7	1.12e-03	1.11e-04	3.20e-05	2.35e-05	1.0
Equateur	45.8	9.79e-04	1.14e-04	5.08e-05		
Haut Katanga	49.0	1.23e-03	1.17e-04	3.70e-05		
Haut Lomami	49.0	1.23e-03	1.17e-04	3.70e-05		
Haut Uele	39.7	1.12e-03	1.11e-04	3.20e-05		
Ituri	39.7	1.12e-03	1.11e-04	3.20e-05		
Kasai	53.0	8.02e-04	1.52e-04	4.79e-05		
Kasai Central	53.0	8.02e-04	1.52e-04	4.79e-05		
Kasai Oriental	46.5	1.05e-03	1.02e-04	4.43e-05		
Kinshasa	36.1	5.56e-04	1.05e-04	2.49e-05		
Kongo Central	43.6	1.62e-03	1.08e-04	3.27e-05		
Kwango	42.4	9.08e-04	9.54e-05	2.35e-05		
Kwilu	42.4	9.08e-04	9.54e-05	2.35e-05		
Lomami	46.5	1.05e-03	1.02e-04	4.43e-05		
Lualaba	49.0	1.23e-03	1.17e-04	3.70e-05		
Mai Ndombe	42.4	9.08e-04	9.54e-05	2.35e-05		
Maniema	39.1	1.12e-03	9.23e-05	3.20e-05		
Mongala	45.8	9.79e-04	1.14e-04	5.08e-05		
Nord Kivu	40.9	8.73e-04	4.89e-05	1.72e-05		
Nord Ubangi	45.8	9.79e-04	1.14e-04	5.08e-05		
Sankuru	46.5	1.05e-03	1.02e-04	4.43e-05		
Sud Kivu	48.8	1.66e-03	1.43e-04	3.56e-05		
Sud Ubangi	45.8	9.79e-04	1.14e-04	5.08e-05		
Tanganyika	49.0	1.23e-03	1.17e-04	3.70e-05		
Tshopo	39.7	1.12e-03	1.11e-04	3.20e-05		
Tshuapa	45.8	9.79e-04	1.14e-04	5.08e-05		

Immunity

Immunity to rubella was assumed to be binary (i.e., either present or absent). Naturally derived immunity (i.e., infection) was assumed to provide life-long protection and did not wane. In relevant simulations, vaccine derived immunity was introduced through both routine immunization (RI) and supplementary immunization activities (SIA)s. Agents also received on-birth immunity that waned as a step function.

All immunity processes (i.e., maternal protection, routine immunization, and supplemental immunization activities) were evaluated after birth and aging, but before disease and mortality processes.

Maternal Protection

Maternally derived rubella antibodies provided a mean of 3 months immunity [4]. All agents were assigned a failure-age (a_f) in days, drawn randomly from the distribution given in Equation 1, that maternally derived protection ended.

$$P\left(a_f\right) = \frac{\exp\left(\frac{a_{f_0}-a_f}{r}\right)}{r\left(\exp\left(\frac{a_{f_0}-a_f}{r}\right)+1\right)^2} \qquad (1)$$

This distribution is characterized by a median failure-age $a_{f_0} = 90$ days, and distribution width $r = 30$ days. The distribution width is interpretable as the mean half-life of maternal antibodies. Calculated failure-ages represent the age at which maternal antibodies wane below the concentration threshold for protection from clinical rubella infection.

Routine Immunization

For those simulations including routine immunization, all agents born in or after simulation year 2021 were also assigned an age in days, drawn randomly from a Gaussian distribution, for routine immunization. Parameters for this distribution were constant across all provinces: $\mu = 300$ days, $\sigma = 90$ days.

The probability of receiving a vaccination at the assigned age through routine services, $\mathrm{RI}_{\mathrm{prob}}$, varied by province. These probabilities are given in Table 3.

Values in Table 3 were taken from the 2018 DRC Multiple Indicator Cluster Survey [2] (MICS). If an individual agent's age at routine immunization exceeded its respective age when maternal protection ended, the agent received immunity from the vaccination, otherwise no immunity was given.

Table 3: Probabilities of receiving vaccine through RI.

Province	RI_{prob}
Bas Uele	0.437
Equateur	0.519
Haut Katanga	0.655
Haut Lomami	0.542
Haut Uele	0.600
Ituri	0.689
Kasai	0.298
Kasai Central	0.753
Kasai Oriental	0.420
Kinshasa	0.759
Kongo Central	0.686
Kwango	0.478
Kwilu	0.544
Lomami	0.422
Lualaba	0.422
Mai Ndombe	0.377
Maniema	0.221
Mongala	0.319
Nord Kivu	0.801
Nord Ubangi	0.389
Sankuru	0.389
Sud Kivu	0.733
Sud Ubangi	0.431
Tanganyika	0.358
Tshopo	0.298
Tshuapa	0.349

Supplemental Immunizations

Additional opportunities for vaccination beyond RI services are provided by SIAs. In practice, SIAs within a province occur over a period of about several weeks and target all children within a specified age range. For simplicity, the model represents these activities as happening everywhere within a province on a single day. Given that an agent's age fell within an SIA's targeted age range, the agent received a vaccination with a specified probability (i.e., the SIA's coverage).

Vaccine delivered via SIA respects maternal protection in the same manner as RI doses. If an individual agent's age when receiving a vaccine was greater than its respective age when maternal protection ended, the agent received immunity from the vaccination, otherwise no immunity was given.

Disease

Rubella infections were represented by an incubation period followed by an infectious period. The duration of the incubation period was drawn randomly from a Gaussian distribution with mean $\mu_{incub} = 17$ days and standard deviation $\sigma_{incub} = 2$ days; the duration of the infectious period was drawn randomly from a Gaussian distribution with mean $\mu_{infect} = 6$ days and standard deviation $\sigma_{infect} = 2$ days.

Agents could only receive one infection. At the end of the infectious period, agents received permanent immunity to subsequent infection. Disease processes were evaluated subsequent to all immunity processes, but before the mortality process.

Infectivity

Each infectious agent (i.e., infected and post-incubation period) was assigned an infectivity drawn randomly from an exponential distribution with mean β, the base infectivity. The basic reproductive number, R_0, is equal to β multiplied by the mean infectious period, μ_{infect}.

A non-zero value, β_{min} was also added to the infectivity in all nodes for all time steps, ensuring that if local elimination did occur (i.e., zero infectivity contribution from infected agents), the probability of infection remained non-zero. This value was used to represent the risk of imported infections, and was proportional to both the population and the base infectivity:

$$\beta_{min} = b_1 \frac{\text{Pop}}{100\text{k}} \beta \tag{2}$$

Proportionality to base infectivity ensured a constant rate of imported infections regardless of the equilibrium susceptibility level. A fixed value of $b_1 = 0.05$ was used in all simulations; this value corresponds to approximately one externally forced infection every two-week period per million population.

The total amount of infectivity present in a node at a given time step was used to determine the number of infections in that node on the subsequent time step. The number of infection events occurring in a node was drawn randomly from a Poisson distribution with rate parameter equal to the total infectivity present in that node at the end of the previous time step. The selection of agents within a node for infection was done without respect to age or other individually-varying property (i.e., homogeneous, well-mixed nodes). The selection was made with replacement, although agents were only eligible to receive a single infection and subsequent infections were ignored.

References

[1] Ministry of Planning and Monitoring of the Implementation of the Modern Revolution - Congo, Ministry of Public Health - Congo, and ICF International. Democratic republic of congo demographic and health survey (edsrdc) 2013-2014. Technical report, MSP - Congo, MPSMRM - Congo, and ICF International, Rockville, Maryland, USA, 2014.

[2] National Institute of Statistics and United Nations Children's Fund. Multiple Indicator Cluster Survey in the DRC, 2018. Technical report, NIS and UNICEF, 2018.

[3] UN OCHA Country office in Democratic Republic of Congo. DR Congo - Health Zones.

[4] S. Waaijenborg, S. J. M. Hahné, L. Mollema, G. P. Smits, G. A. M. Berbers, F. R. M. van der Klis, H. E. de Melker, and J. Wallinga. Waning of Maternal Antibodies Against Measles, Mumps, Rubella, and Varicella in Communities With Contrasting Vaccination Coverage. *The Journal of Infectious Diseases*, 208(1):10–16, 05 2013.

Examination of scenarios introducing rubella vaccine in the Democratic Republic of the Congo

Additional File 2 - Infectivity Calibration and Burden Estimation

Kurt Frey

Infectivity of a disease can be characterized by its basic reproductive number, R_0, which describes the number subsequent infections that a single case would generate in an otherwise uninfected population. This property is a consequence of both the biology of the pathogen and the social network structure of the at-risk population. Accurate estimation, R_0, is important because it also describes the immunity level necessary to control the spread of infection with that population.

Overview

Simulations used to estimate the infectivity of rubella in the provinces of the Democratic Republic of the Congo (DRC) had a duration of 51 years, which was taken to represent the period from 1970 to 2020. Simulations used to estimate the burden of congenital rubella syndrom (CRS) had a duration of 81 years, which was taken to represent the period from 1970 to 2050. Results for the period 1970 to 2010 were not intended to be an accurate historical reproduction. Those time steps were used to provide an equilibration period so that the period from 2011 onward would not be biased by the choice of initial conditions.

Serosurvey Data

Serosurvey data was collected between November 2013 and February 2014 as part of the second Demographics and Health Survey (DHS) conducted in the DRC [2]. Weighted counts of seronegative observations and total observations are given in Table 1. No values were recorded for the age range 5 to 15 years old.

Infectivity Calibration

Age-structured susceptibility profiles for the simulated DRC provinces were recorded using the same age binning as the reference data. For infectivity calibration, each simulation recorded monthly histograms over 10 simulated years (2011 to 2020), for a total of 120 susceptibility histograms per province for each simulation. Outcomes for each province were independent. A total of 2500 simulations outcomes for each province were used.

Objective Function

A Gaussian likelihood function, Equation 1, was used to score each simulated susceptibility histogram.

$$\max_{x>0} L(x) = \prod_{\text{age}} \frac{1}{x\sigma_{\text{age}}\sqrt{2\pi}} e^{-\frac{1}{2}\left(\frac{\mu_{\text{age,obs}}-\mu_{\text{age,sim}}}{x\sigma_{\text{age}}}\right)^2} \quad (1)$$

For a given province, each of the 12 age categories from Table 1 was characterized by $\mu_{\text{age,obs}}$, the observed fraction of seronegativity, and $\mu_{\text{age,sim}}$, the simulated fraction of seronegativity. The variance in each age category, σ^2_{age} was set equal to the weighted total counts for that that age category and province. A single variance inflation parameter, x, applied to all age categories for a given province and simulation. Each province was evaluated independently. Each of the simulated histograms was compared with observed serosurvey data, and the maximum value from among the 120 simulated histograms was retained as the representative value for a given province in that simulation.

Posterior Distribution

Pre-calibration base infectivity, β, was drawn randomly from a uniform distribution over the range [0.25, 2.50]. This range corresponds to sampling R_0 over the range [1.5, 15.0]. Outcomes from 2500 simulations were used to construct posterior distributions. Simulations provide independent results for each province.

Results for Bas Uele province are shown in Figure 1 as an example. Each point in Figure 1 is the maximum outcome for the 120 histograms generated by a simulation and scored using the Gaussian likelihood function. The likelihood value for Bas Uele province was maximized between 0.50 and 0.75 (corresponding to an R_0 between 3.0 and 4.5.

In each province, the 2500 outcome values were correlated using a Gaussian kernel density estimator, with the likelihood

Table 1: Reference data for rubella antibody detection from the 2013 DHS in the DRC. Numerator values are the weighted number of individuals without rubella antibodies (seronegative individuals) and denominator values are the weighted number of individuals tested in total (total individuals).

Province	Seronegativity (weighted counts; bin ages in years)											
	0.5 - 1	1 - 2	2 - 3	3 - 4	4 - 5	15 - 20	20 - 25	25 - 30	30 - 35	35 - 40	40 - 45	45 - 50
Bas Uele	$\frac{12.14}{13.42}$	$\frac{27.32}{39.99}$	$\frac{18.66}{30.01}$	$\frac{23.71}{37.88}$	$\frac{14.12}{29.82}$	$\frac{9.86}{54.37}$	$\frac{2.43}{50.96}$	$\frac{6.89}{59.71}$	$\frac{0.00}{47.99}$	$\frac{7.07}{48.82}$	$\frac{0.56}{19.50}$	$\frac{2.03}{13.35}$
Equateur	$\frac{18.77}{19.63}$	$\frac{53.77}{66.56}$	$\frac{23.80}{34.64}$	$\frac{29.80}{54.89}$	$\frac{12.56}{34.24}$	$\frac{5.20}{119.62}$	$\frac{3.26}{60.51}$	$\frac{6.07}{92.32}$	$\frac{0.82}{51.75}$	$\frac{0.00}{41.29}$	$\frac{12.74}{84.92}$	$\frac{4.67}{15.38}$
Haut Katanga	$\frac{26.39}{32.86}$	$\frac{32.94}{57.33}$	$\frac{52.94}{93.01}$	$\frac{20.96}{48.52}$	$\frac{15.50}{63.31}$	$\frac{0.76}{141.83}$	$\frac{1.23}{134.25}$	$\frac{0.00}{96.63}$	$\frac{0.00}{94.32}$	$\frac{0.87}{80.07}$	$\frac{0.00}{24.17}$	$\frac{0.99}{54.67}$
Haut Lomami	$\frac{19.25}{22.64}$	$\frac{32.51}{42.28}$	$\frac{28.41}{33.62}$	$\frac{24.88}{39.97}$	$\frac{24.99}{31.90}$	$\frac{5.15}{84.85}$	$\frac{1.45}{62.31}$	$\frac{1.67}{60.18}$	$\frac{0.00}{52.87}$	$\frac{1.34}{34.68}$	$\frac{0.00}{32.62}$	$\frac{0.43}{23.55}$
Haut Uele	$\frac{17.23}{17.94}$	$\frac{27.20}{27.80}$	$\frac{19.01}{20.88}$	$\frac{23.98}{24.57}$	$\frac{8.08}{11.39}$	$\frac{4.04}{36.70}$	$\frac{11.67}{76.35}$	$\frac{12.64}{50.04}$	$\frac{0.52}{49.40}$	$\frac{2.21}{25.53}$	$\frac{0.00}{14.32}$	$\frac{1.38}{20.89}$
Ituri	$\frac{17.00}{22.99}$	$\frac{36.09}{56.68}$	$\frac{26.03}{32.24}$	$\frac{27.75}{47.11}$	$\frac{19.20}{36.79}$	$\frac{5.56}{108.10}$	$\frac{7.30}{81.80}$	$\frac{2.80}{71.25}$	$\frac{0.00}{64.52}$	$\frac{3.01}{59.07}$	$\frac{1.50}{41.15}$	$\frac{2.74}{43.27}$
Kasai	$\frac{19.18}{27.13}$	$\frac{47.73}{58.89}$	$\frac{37.32}{50.84}$	$\frac{37.21}{56.04}$	$\frac{24.20}{38.32}$	$\frac{13.11}{105.29}$	$\frac{13.16}{85.99}$	$\frac{9.30}{57.70}$	$\frac{0.00}{57.11}$	$\frac{2.14}{39.35}$	$\frac{0.00}{29.06}$	$\frac{0.54}{50.84}$
Kasai Central	$\frac{39.76}{50.51}$	$\frac{44.52}{55.93}$	$\frac{60.06}{88.88}$	$\frac{40.27}{53.25}$	$\frac{30.88}{60.03}$	$\frac{14.60}{146.51}$	$\frac{10.88}{117.85}$	$\frac{5.18}{99.95}$	$\frac{2.03}{69.58}$	$\frac{1.44}{78.06}$	$\frac{2.66}{49.70}$	$\frac{13.82}{59.92}$
Kasai Oriental	$\frac{22.91}{27.93}$	$\frac{70.02}{78.05}$	$\frac{56.22}{67.79}$	$\frac{39.48}{52.34}$	$\frac{39.59}{59.49}$	$\frac{12.49}{158.36}$	$\frac{2.97}{103.74}$	$\frac{12.40}{98.60}$	$\frac{12.85}{85.87}$	$\frac{0.73}{54.96}$	$\frac{0.46}{52.25}$	$\frac{3.17}{44.48}$
Kinshasa	$\frac{50.62}{55.12}$	$\frac{81.35}{115.11}$	$\frac{66.01}{118.84}$	$\frac{47.68}{78.42}$	$\frac{42.83}{108.84}$	$\frac{27.95}{330.34}$	$\frac{16.60}{365.24}$	$\frac{13.64}{287.52}$	$\frac{9.51}{201.67}$	$\frac{3.26}{163.60}$	$\frac{2.39}{177.16}$	$\frac{3.03}{112.96}$
Kongo Central	$\frac{32.73}{39.33}$	$\frac{49.43}{71.21}$	$\frac{31.05}{60.47}$	$\frac{23.99}{57.26}$	$\frac{36.34}{77.99}$	$\frac{14.39}{140.46}$	$\frac{21.92}{100.42}$	$\frac{7.79}{116.82}$	$\frac{4.85}{57.12}$	$\frac{2.12}{65.59}$	$\frac{0.00}{52.13}$	$\frac{3.32}{44.69}$
Kwango	$\frac{27.65}{29.78}$	$\frac{38.78}{73.51}$	$\frac{29.66}{80.30}$	$\frac{34.80}{85.80}$	$\frac{17.68}{73.52}$	$\frac{13.29}{150.08}$	$\frac{3.55}{96.55}$	$\frac{5.12}{113.67}$	$\frac{5.85}{100.27}$	$\frac{2.19}{103.03}$	$\frac{2.45}{78.56}$	$\frac{1.74}{64.23}$
Kwilu	$\frac{42.46}{62.56}$	$\frac{66.13}{124.68}$	$\frac{39.98}{107.14}$	$\frac{50.99}{120.68}$	$\frac{50.52}{111.64}$	$\frac{13.06}{192.34}$	$\frac{18.60}{169.82}$	$\frac{19.78}{170.19}$	$\frac{26.83}{156.09}$	$\frac{1.33}{106.13}$	$\frac{0.00}{84.21}$	$\frac{0.89}{103.26}$
Lomami	$\frac{28.31}{38.37}$	$\frac{71.23}{78.81}$	$\frac{67.84}{84.91}$	$\frac{56.65}{77.58}$	$\frac{61.09}{72.64}$	$\frac{43.43}{178.95}$	$\frac{9.50}{133.89}$	$\frac{3.11}{106.60}$	$\frac{7.18}{104.96}$	$\frac{0.39}{55.03}$	$\frac{0.00}{39.53}$	$\frac{3.81}{52.21}$
Lualaba	$\frac{13.82}{17.29}$	$\frac{30.77}{32.99}$	$\frac{23.34}{28.27}$	$\frac{15.71}{21.13}$	$\frac{9.79}{20.16}$	$\frac{1.60}{58.00}$	$\frac{0.00}{50.00}$	$\frac{0.62}{48.53}$	$\frac{0.00}{38.54}$	$\frac{0.00}{27.71}$	$\frac{0.23}{23.80}$	$\frac{0.00}{20.48}$
Mai Ndombe	$\frac{23.32}{38.15}$	$\frac{39.97}{70.89}$	$\frac{27.73}{52.54}$	$\frac{44.70}{80.43}$	$\frac{24.57}{77.43}$	$\frac{9.96}{143.47}$	$\frac{5.96}{103.24}$	$\frac{11.35}{144.05}$	$\frac{2.89}{108.22}$	$\frac{1.68}{79.89}$	$\frac{2.74}{69.79}$	$\frac{7.91}{53.23}$
Maniema	$\frac{17.97}{21.80}$	$\frac{38.21}{49.16}$	$\frac{33.16}{52.13}$	$\frac{57.99}{76.74}$	$\frac{27.16}{45.20}$	$\frac{10.29}{93.61}$	$\frac{13.67}{77.85}$	$\frac{6.31}{74.73}$	$\frac{7.21}{69.64}$	$\frac{0.65}{71.00}$	$\frac{1.27}{55.73}$	$\frac{1.73}{39.16}$
Mongala	$\frac{15.83}{28.99}$	$\frac{27.17}{45.93}$	$\frac{34.17}{53.95}$	$\frac{35.38}{55.61}$	$\frac{30.07}{46.85}$	$\frac{11.60}{95.01}$	$\frac{11.30}{43.44}$	$\frac{5.23}{101.81}$	$\frac{1.74}{40.44}$	$\frac{0.00}{54.91}$	$\frac{0.84}{37.36}$	$\frac{3.52}{37.19}$
Nord Kivu	$\frac{39.51}{43.52}$	$\frac{115.47}{139.79}$	$\frac{90.24}{135.62}$	$\frac{79.11}{121.72}$	$\frac{75.00}{144.82}$	$\frac{21.66}{309.17}$	$\frac{10.32}{278.90}$	$\frac{2.53}{188.84}$	$\frac{9.96}{123.23}$	$\frac{7.31}{122.96}$	$\frac{9.53}{99.02}$	$\frac{8.40}{95.31}$
Nord Ubangi	$\frac{6.76}{7.18}$	$\frac{18.70}{21.90}$	$\frac{22.27}{31.05}$	$\frac{11.13}{19.84}$	$\frac{17.39}{24.96}$	$\frac{4.11}{37.88}$	$\frac{5.29}{51.08}$	$\frac{5.22}{50.73}$	$\frac{5.99}{36.16}$	$\frac{1.77}{25.76}$	$\frac{0.00}{28.87}$	$\frac{0.00}{18.41}$
Sankuru	$\frac{12.23}{13.44}$	$\frac{23.13}{28.60}$	$\frac{28.24}{33.19}$	$\frac{21.62}{27.80}$	$\frac{17.24}{22.79}$	$\frac{5.29}{39.28}$	$\frac{5.70}{56.95}$	$\frac{10.15}{47.18}$	$\frac{3.69}{32.54}$	$\frac{5.42}{30.94}$	$\frac{0.00}{14.60}$	$\frac{2.64}{25.23}$
Sud Kivu	$\frac{65.75}{83.99}$	$\frac{86.78}{107.95}$	$\frac{102.07}{121.28}$	$\frac{84.08}{113.37}$	$\frac{57.74}{102.48}$	$\frac{11.82}{166.07}$	$\frac{3.78}{165.33}$	$\frac{0.82}{151.51}$	$\frac{8.56}{131.35}$	$\frac{3.32}{53.35}$	$\frac{0.00}{98.79}$	$\frac{1.77}{76.37}$
Sud Ubangi	$\frac{48.17}{53.68}$	$\frac{55.40}{72.03}$	$\frac{74.29}{85.76}$	$\frac{41.35}{56.26}$	$\frac{59.98}{78.15}$	$\frac{21.04}{155.93}$	$\frac{11.15}{146.50}$	$\frac{1.13}{133.41}$	$\frac{1.89}{80.72}$	$\frac{2.69}{94.23}$	$\frac{0.00}{65.91}$	$\frac{4.01}{53.44}$
Tanganyika	$\frac{14.82}{16.82}$	$\frac{25.89}{32.39}$	$\frac{23.70}{32.66}$	$\frac{21.86}{29.31}$	$\frac{18.84}{26.22}$	$\frac{10.04}{47.67}$	$\frac{9.47}{61.35}$	$\frac{6.48}{52.04}$	$\frac{0.33}{37.86}$	$\frac{0.00}{26.98}$	$\frac{0.51}{20.19}$	$\frac{1.28}{15.10}$
Tshopo	$\frac{16.59}{20.01}$	$\frac{32.19}{43.91}$	$\frac{27.07}{35.05}$	$\frac{27.48}{37.02}$	$\frac{12.63}{24.53}$	$\frac{11.15}{78.10}$	$\frac{11.01}{98.84}$	$\frac{2.63}{61.39}$	$\frac{4.15}{63.30}$	$\frac{4.22}{69.99}$	$\frac{0.00}{24.57}$	$\frac{3.51}{46.56}$
Tshuapa	$\frac{10.00}{11.83}$	$\frac{30.60}{43.30}$	$\frac{24.71}{33.35}$	$\frac{17.97}{25.85}$	$\frac{15.93}{31.70}$	$\frac{0.86}{65.51}$	$\frac{5.24}{63.01}$	$\frac{3.21}{70.86}$	$\frac{4.20}{40.62}$	$\frac{0.94}{42.53}$	$\frac{1.23}{31.03}$	$\frac{1.23}{28.92}$

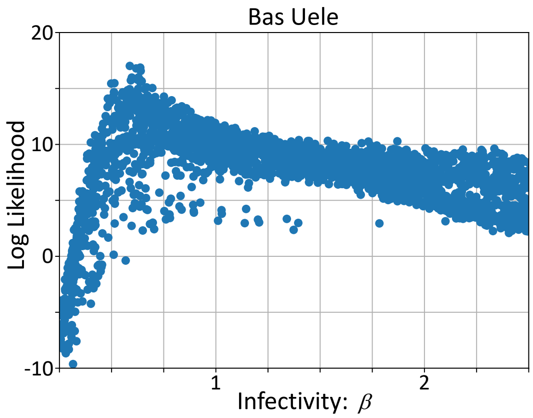

Figure 1: Scatter-plot of simulation outcomes for Bas Uele province. Infectivity was sampled uniformly over the range [0.25, 2.5].

values as the weights for each outcome. These posterior probability densities were used as the input distributions for infectivity in simulations estimating the burden of CRS. The posterior distribution for infectivity in Bas Uele province is shown in Figure 2 as an example.

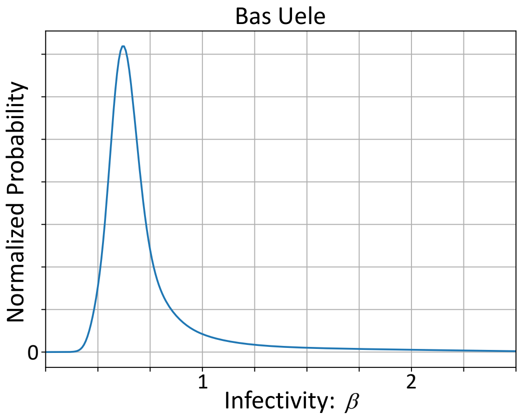

Figure 2: Normalized posterior probability density function for infectivity in Bas Uele province.

Burden Estimation

Age structured infection time-series for the simulated DRC provinces were recorded using the same age binning as the reference data. For CRS burden estimation, each simulation recorded annual, age-structures infections over 30 simulated years (2021 to 2050). Outcomes for each province were independent. A total of 2500 simulations outcomes for each province were used. Infectivity in each province for each of these simulations was drawn randomly from the posterior distributions previously described.

Table 2: Age-specific fertility rates for the three years preceding the 2013 DHS in the DRC.

Province	Fertility (births per 1000 women; ages in years)							
	10 - 15	15 - 20	20 - 25	25 - 30	30 - 35	35 - 40	40 - 45	45 - 50
Bas Uele	7	176	264	255	222	170	75	23
Equateur	8	166	294	332	280	221	94	22
Haut Katanga	2	179	293	350	301	269	134	29
Haut Lomami	2	179	293	350	301	269	134	29
Haut Uele	7	176	264	255	222	170	75	23
Ituri	7	176	264	255	222	170	75	23
Kasai	9	148	379	388	292	331	98	9
Kasai Central	9	148	379	388	292	331	98	9
Kasai Oriental	2	144	329	314	303	256	79	40
Kinshasa	0	67	142	233	185	139	68	0
Kongo Central	0	172	263	299	204	176	62	21
Kwango	5	126	272	304	283	197	86	1
Kwilu	5	126	272	304	283	197	86	1
Lomami	2	144	329	314	303	256	79	40
Lualaba	2	179	293	350	301	269	134	29
Mai Ndombe	5	126	272	304	283	197	86	1
Maniema	1	161	286	318	196	181	111	0
Mongala	8	166	294	332	280	221	94	22
Nord Kivu	1	103	242	273	296	209	147	32
Nord Ubangi	8	166	294	332	280	221	94	22
Sankuru	2	144	329	314	303	256	79	40
Sud Kivu	1	128	346	349	287	246	165	15
Sud Ubangi	8	166	294	332	280	221	94	22
Tanganyika	2	179	293	350	301	269	134	29
Tshopo	7	176	264	255	222	170	75	23
Tshuapa	8	166	294	332	280	221	94	22

Fertility

Age structured fertility rates for the DRC provinces were taken from the 2013 DHS [2]. These values have been reproduced in Table 2.

Congenital Rubella Syndrome

Fertility values were converted into a probability of pregnancy for each age group. Given N pregnancies per-1000-women per-36-months (Table 2), the probability of a randomly selected individual being pregnant is $0.5*9*N/36/1000$, or N in 8000. This calculation assumes 9 month pregnancies and equal numbers of men and women. Further assuming no bias in infection due to gender or pregnancy status, total infection numbers for each age group were converted into infections-while-pregnant.

Not all rubella infections during pregnancy result in fetal death, stillbirth, or birth defects; infections during early pregnancy cause the greatest harm [1]. However, a fixed ratio of 50% was assumed when converting infections-while-pregnant into CRS burden. All adverse outcomes, including fetal death and stillbirth, were grouped together as occurrences of CRS burden.

References

[1] E. Miller, J. Cradock-Watson, and T. Pollock. Consequences of confirmed maternal rubella at successive stages of pregnancy. *The Lancet*, 320(8302):781 – 784, 1982. Originally published as Volume 2, Issue 8302.

[2] Ministry of Planning and Monitoring of the Implementation of the Modern Revolution - Congo, Ministry of Public Health - Congo, and ICF International. Democratic republic of congo demographic and health survey (eds-rdc) 2013-2014. Technical report, MSP - Congo, MPSMRM - Congo, and ICF International, Rockville, Maryland, USA, 2014.

Examination of scenarios introducing rubella vaccine in the Democratic Republic of the Congo

Additional File 3 - Sub-provincial Spatial Heterogeneity

Kurt Frey

Length scale within simulations of disease transmission is a consequence of model selection. Increasing the geographic resolution (e.g., from national to provincial to district or zone) decreases that length scale and may change outcomes depending on the parameters describing long distance connections. Selection of resolution and connectivity parameters is important because of the data required to inform parameter values.

Overview

Provinces of the Democratic Republic of the Congo (DRC) were simulated as homogeneously well-mixed internally with respect to transmission dynamics, and independent of one another with respect to long distance connections. This approximation implies that the location of individuals within a province was not estimated and properties were uniform throughout the province. This level of resolution was appropriate for the serosurvey data used to infer rubella infectivity.

Internal Homogenity

Serosurvey data was collected between November 2013 and February 2014 as part of the second Demographics and Health Survey (DHS) conducted in the DRC [1]. This survey used cluster sampling to estimate population statistics, and the internal homogenity of the clusters can be quantified using beta binomial likelihood maximization.

In the homogeneous limit, the probability of a seronegative result in each individual cluster is equal to mean probability of a seronegative result across all clusters. The heterogeneous limit for this system has clusters at the extremes of either 0% or 100% seronegative with probability equal to the mean probability of a seronegative result across all clusters.

Objective Function

A beta binomial likelihood function was used to score the distribution of clusters in each province. Only data for seronegativity in the 6mo to 5yr age cohort was examined. The cohort was subdivided into five age categories (6mo-1yr, 1yr-2yr, 2yr-3yr, 3yr-4yr, and 4yr-5y) with independent means, p_{age}, for each category. A single homogeneity parameter, X, was applied to all age categories and represented the level of homogeneity for the province. Parameters to the beta binomial used $\alpha = (p_{age})X$ and $\beta = (1 - p_{age})X$.

Table 1: Values of the homogeneity parameter, indicating the degree of uniformity across clusters within a province.

Province	Homogenity
Bas Uele	3.6
Equateur	9.7
Haut Katanga	99.9
Haut Lomami	8.0
Haut Uele	99.9
Ituri	4.0
Kasai	3.0
Kasai Central	6.4
Kasai Oriental	99.9
Kinshasa	99.9
Kongo Central	3.7
Kwango	4.0
Kwilu	9.4
Lomami	7.5
Lualaba	11.6
Mai Ndombe	11.6
Maniema	12.2
Mongala	37.8
Nord Kivu	7.8
Nord Ubangi	15.7
Sankuru	4.1
Sud Kivu	5.2
Sud Ubangi	99.9
Tanganyika	10.4
Tshopo	4.8
Tshuapa	6.8

In this formulation, the homogeneous limit (i.e., binomial distribution) is approached for very large values of the homogeneity parameter, X. A zero value corresponds to the heterogeneous limit. The transition between regimes occurs at $X = 2.0$; at the transition, individual clusters are equally likely to demonstrate either mean probability (p_{age}) or extreme probability (0% or 100%).

Province Outcomes

Maximum likelihood of the homogeneity parameter in all provinces was greater than 2.0; these values are provided in Table 1. Possible values for the homogeneity parameter were restricted to the range (0.01, 100.0).

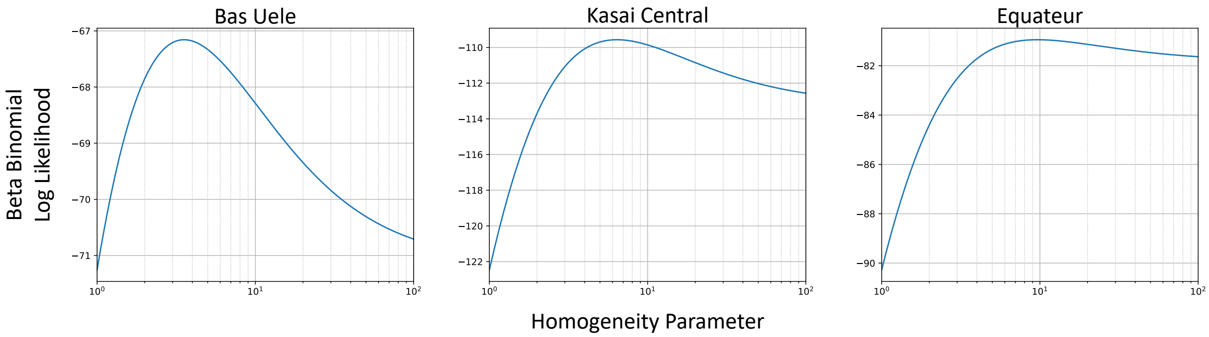

Figure 1: Likelihoods for homogeneity parameter values in Bas Uele, Kasai Central, and Equateur provinces.

Outcomes have been qualitatively grouped based on the values of the homogeneity parameter. Values > 9.0 indicate no evidence of heterogeneity, values between 5.0 and 9.0 have weak evidence of heterogeneity, and values < 5.0 are stronger signals of heterogeneity. Using these classifications, half of the provinces did not have evidence of heterogeneity, a quarter had weak evidence, and a quarter had strong evidence.

Figure 1 contrasts the homogeneity parameter likelihoods in Bas Uele, Kasai Central, and Equateur provinces. Maximum likelihood parameter values in these three provinces were 3.6, 6.4, and 9.7, respectively, and correspond to the qualitatively strong, weak, and no heterogeneity classifications.

Heterogeneity was generally low, supporting the use of a well mixed assumption at the province level. Evidence of sub-provincial heterogeneity was present in several provinces, although this heterogeneity did not constitute a high degree of clustering from the perspective of disease transmission.

A high degree of clustering (homogeneity parameter < 2.0) would correspond to clusters of extreme seronegativity (0% or 100%) suggesting localized outbreaks and pockets of susceptibility. Data from the second DHS in the DRC do not support this interpretation.

References

[1] Ministry of Planning and Monitoring of the Implementation of the Modern Revolution - Congo, Ministry of Public Health - Congo, and ICF International. Democratic republic of congo demographic and health survey (eds-rdc) 2013-2014. Technical report, MSP - Congo, MPSMRM - Congo, and ICF International, Rockville, Maryland, USA, 2014.

Examination of scenarios introducing rubella vaccine in the Democratic Republic of the Congo

Additional File 4 - Outcomes for supplemental immunization activities with limited effectiveness

Kurt Frey

Scenarios introducing rubella containing vaccine (RCV) to the Democratic Republic of the Congo (DRC) examined implementing supplemental immunization activities (SIAs) at various levels of effectiveness and frequencies. The most pessimistic of these scenarios involved SIAs occurring once every four years and achieving around 50% coverage; given current levels of routine immunization services for measles, this frequency and coverage would be inadequate for measles control and is a credible lower bound for rubella vaccine usage.

Overview

Outcomes for the artificial context described in section 3.4 of the main text were re-implemented to also include SIAs every four years; SIAs achieved around 50% coverage. These simulations contrast artificial context simulations from the main text, which estimate congenital rubella syndrome (CRS) burden at various routine immunization levels absent any SIAs. Outcomes presented in this supplement are qualitatively similar to results depicted in Figure 3 of the main text, although outcomes here are not specific to any particular province. The artificial context uses mean provincial birth and population growth rates, along with infectivity sampled from a posterior distribution with mean R_0 of around 5.

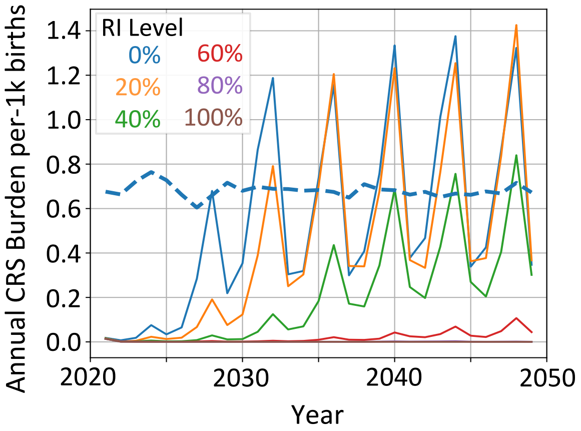

Figure 1: Mean annual CRS burden per thousand births as a function of time following RCV introduction through RI and SIAs. Dashed line provides a no-vaccine reference.

Burden Estimates

Vaccine introduction that incorporates an initial catch-up SIA targeting an age range from 9mo to 15yrs, and subsequent follow-up SIAs every four years that target an age range from 9mo to 5yrs, results in a period of nearly a decade with significantly reduced CRS burden. These outcomes are depicted in figure 1; the dashed line provides a no-vaccine reference for burden. The initial period of reduced burden occurs even without ongoing RI services, but reduced burden cannot be maintained absent those services.

Inverse Response in CRS Burden

Total rubella infections decrease for all scenarios examined, as depicted in figure 2. Contrasting figures 1 and 2 emphasizes that the inverse response in CRS burden (increasing CRS with decreasing rubella infections) occurs only at lower levels of coverage and several years post-introduction.

Vaccination through SIAs in these scenarios increases effective total coverage by about 50%, and is not correlated with routine immunization services. Total coverage levels for the RI levels are about 50% total for no RI, 60% total for 20% RI, etc.

Stochastic Behavior

Several years post-vaccine introduction, mean CRS burden can increase when immunization services are insufficient to maintain local elimination; however, the distribution of this burden in time also changes. Figure 3 depicts the histogram of simulated outcomes over the period 2040 to 2050 for the no-vaccine reference trajectory and 20% RI levels (blue dashed line and solid orange line respectively from figure 1).

Although mean burden may increase in the inter-SIA period,

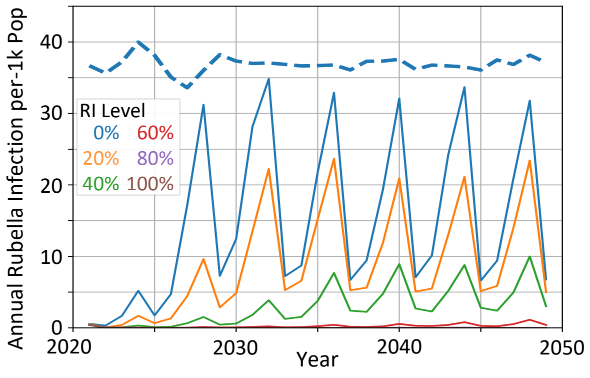

Figure 2: Mean annual rubella infections per thousand population as a function of time following RCV introduction through RI and SIAs. Dashed line provides a no-vaccine reference.

this increase in mean burden is realized though a greatly increased risk of large outbreaks and not through any deterministic, periodic behavior. The most likely outcome during this inter-SIA period is near-zero burden; importations that may circulate locally prior to fade-out, but that do not lead to large outbreaks. Most simulations at this level of vaccination do not generate large, susceptible-depleting outbreaks during the 30-year simulation window.

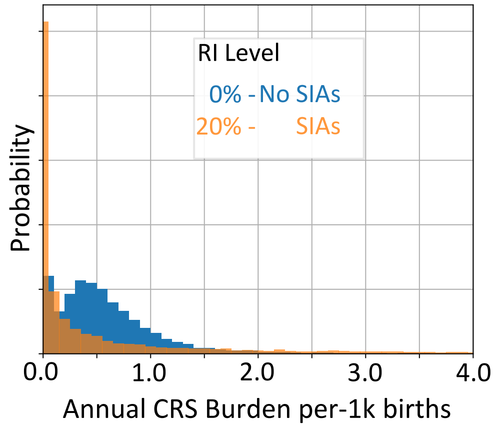

Figure 3: Histogram of simulated annual CRS burden over the period 2040 to 2050; outcomes are shown for the zero RI / no SIA scenario (reference) and the scenario with 20% RI and SIAs every fourth year.

Pre-vaccine trajectories also incorporate this asymmetry in annual burden distribution, but to a much lesser degree. The saw-tooth patterns depicted in figure 3 are best interpreted as a risk metric and not precise burden forecasts. Large outbreaks can occur in the year following an SIA, although with lower probability. Details of the importation rate are presented in additional file 1.

Relation to Province Outcomes

Given the SIA schedule implemented in these simulations, the transition between long-term CRS burden reduction and potential burden increases occurs at a RI level of 40%. This level is a consequence of the assumed demographics and infectivity, and should not be interpreted as a precise threshold. In the main text, this SIA scenario applied to the province of Haut Katanga results in roughly equivalent burden to the no-vaccine scenario (estimated RI level of around 60% in Haut Katanga). That equivalence is due in large part to the elevated level of infectivity estimated for that province (mean R_0 of around 7).

Estimated rubella infectivity has a large impact on projected burden. More populous provinces like Haut Katanga and Kinshasa were estimated to have higher infectivity levels because their large populations were more likely to support endemic transmission (in contrast to less populous provinces where high levels of seropositivity in serosurvey results implied recent outbreaks).

Higher levels of estimated rubella infectivity are not contraindicative for vaccine introduction. Locations with high levels of infectivity are also expected to have (comparatively) lower levels of CRS rates pre-vaccine introduction because immunity will tend to be naturally acquired via infection at a young age. Post-vaccine introduction these regions will be at elevated risk of resurgence due to importation, but are still expected to have reductions in median burden.
